# A blockchain secured metaverse framework for scalable and immersive telemedicine

**DOI:** 10.1038/s41598-025-12068-6

**Published:** 2025-07-22

**Authors:** Rahul Ganpatrao Sonkamble, Swati Shirke-Deshmukh, Vijay Katkar, M. Lunagaria, Ganshyam G. Tejani, Seyed Jalaleddin Mousavirad

**Affiliations:** 1 Department of Computer Science & Engineering, Pimpri Chinchwad University, Pune, Maharashtra India; 2 Department of Computer Engineering, Gujarat Technological University, Ahmedabad, 382424 Gujarat India; 3 Department of Research Analytics, Saveetha Institute of Medical and Technical Sciences, Saveetha Dental College and Hospitals, Saveetha University, Chennai, 600077 India; 4 Department of Industrial Engineering and Management, Yuan Ze University, Taiwan, 320315 Jordan; 5 Department of Computer and Electrical Engineering, Mid Sweden University, Sundsvall, Sweden

**Keywords:** Mathematics and computing, Applied mathematics, Computational science

## Abstract

The rapid evolution of telemedicine has enhanced healthcare accessibility, yet significant challenges persist, particularly in data security, patient engagement, latency, and scalability. Existing telemedicine solutions rely on centralized architectures, making Electronic Health Records (EHRs) susceptible to data breaches and unauthorized access. This research proposes a novel system which integrates the metaverse and blockchain into telemedicine which can be a transformative approach to solve problems in remote healthcare. By combining immersive virtual environments with decentralized data management, the proposed solution described in this paper aims to give users more ways to interact with each other, enhanced data security, increased efficiency, and higher scalability. The Metaverse serves as the foundation for the implementation of 3D consultation rooms, virtual training spaces, and individual care. Blockchain offers safe, transparent, and immutable data exchange that will create patient-empowered medical records for them. Real-time devices and analysis of real-time physiological data from wearables, sensors, Internet of Things (IoT) devices, and Artificial Intelligence (AI) analytics complete the system. The proposed solution extensively uses Virtual Reality (VR)/Augmented Reality (AR) devices, IoT sensors, Ethereum, and the Unity 3D platform, among others. Assessments indicate that system receives a significantly high level of satisfaction from its users, better secured data, increased automation of processes, and compliance with global standards such as General Data Protection Regulation (GDPR). Compliance with such global standards is achieved through smart contract-based access management, smart contract-based consent management, and immutable audit trails in the blockchain. Moreover, this research demonstrates that incorporating high-tech tools like AI and VR into telemedicine is currently feasible. This paves the way for the creation of even more secure and user-friendly telemedicine platforms that employ neural networks. This research sets a foundation for next-generation telemedicine ecosystems.

## Introduction

The current progressive advent of information technology is revolutionizing the global healthcare system, particularly through telehealth^1,2^. Integrating Metaverse with blockchain presents a promising way to address challenges of telemedicine by facilitating the secure, interactive, and immersive healthcare environment^3^. Such integration of these technologies will foster reliable, open, and interactive systems that will help patients to deliver accurate and secure health information.

With features as three-dimensional, immersive, and an interactive virtual space, the metaverse provides an ability to collaborate in real time in a digital space^4^. For healthcare providers the metaverse can provide avatar-to-avatar physical consults, develop models for patient information delivery and even includes surgeries in the simulation for instructional purposes^5^. The use of such environments appears to present an opportunity to overcome many barriers associated with patients in hard-to-reach areas availing themselves for the needed healthcare services. Studies have further indicated that, telecommunication in patient involvement enhances patient satisfaction since they practice real life situations in virtual frameworks^6^.

Nonetheless, traditional telemedicine has faced severe barriers which include data privacy infringement^7,8^, lack of trust in patient’s care, and the limited interpersonal touch that accompanies face-to-face consultation. There might be the cases of deepfake as well.

This is where blockchain technology plays a transformative role. As a decentralized and a non-tamperable ledger system, blockchain offers secure data, privacy preservation, and transparency^9^. It maintains the records of patients through encryption keys for ciphering, which ensures that only authorized stakeholders should have access to health information. Also, consent management and insurance claims are automated by smart contracts, significantly reducing the workload and streamlining healthcare administration^10^.

Integration of the metaverse and blockchain is a perfect fit. While the metaverse improves the practicality of telemedicine by increasing its interactivity, blockchain provides the appropriate security needed for telemedicine to become more deeply integrated into society. For instance, general practice consultations via avatars in the metaverse may be integrated with blockchain Electronic Health Record (EHRs) where clinician can access patient records without the compromise of the EHR while preserving the mediated record^11^. Down this line, such integration of the technologies is almost limitless with several possible implementations. Consider a virtual hospital where the patients may use tele-pharmacy^12^, teleconsultations^13^, teletherapy^14^, and even tele-physiotherapy^15^. All their data are protected under smart contracts on the blockchain. Also, teaching and training medical people can occur in realistic simulations in the metaverse, thus cutting on costs of physical resources for training people. This constantly increasing platform makes sure that certifications and training credentials cannot be forged or changed in any way.

### Organization of the paper

The remainder of this paper is organized as follows. The Sect. "Identified research gaps" points out research gaps and challenges in existing telemedicine systems related to data privacy, scalability, and the user experience. Section "Challenges in traditional telemedicine" specifically describes the limitations of standard telemedicine and justifies integration of blockchain and metaverse. The proposed framework stated in Sect. "Proposed model overview" comprises layers, smart contracts based access and consent management, and system interaction across all the components. The Sect. "Literature survey" is organised to summarize the evolution, economic challenges, advantages, similarities and metaverse-related advancements in existing telemedicine approaches. Section "System architecture and layered interaction" describes implementation details and simulation-based performance evaluations of the system, based on latency, scalability, user satisfaction, and accessibility. Section "Experimentation and results" highlights how the proposed system is better than recent state-of-the-art works regarding efficiency, security and interoperability. In the Sect. "Conclusion", concluding remarks have been given. Finally, the paper concludes with Sect. 9 which briefs limitations and future work for telemedicine sector.

## Identified research gaps

Telemedicine has emerged as a critical enabler for remote healthcare delivery, especially during the COVID-19 pandemic. However, several critical challenges persist:

- **Data Security and Privacy**: Considering that the EHRs are stored centrally, EHRs are at high risk of being hacked, leaked and accessed by unauthorized personnel^16^.
- **Patient Engagement**: Interpersonal touch and contact are characteristic of face-to-face practice and regularly lead to reduced patient satisfaction in telemedicine^17,18^.
- **Scalability**: The advancement in the use of telemedicine has few shortcomings in the corresponding infrastructure where all the patients may not be attended by the doctors in less time^18^.
- **Compliance**: Following cross-border legal regulations like Health Insurance Portability and Accountability Act (HIPAA)^10^ and General Data Protection Regulation (GDPR)^10^ are complex for telemedicine providers.
- **Cost Efficiency**: Although building the blockchain is initially expensive, maintaining traditional systems becomes more expensive with each manual process and repeated security audits.

This research addresses these gaps by integrating the blockchain for secured data and Metaverse for visual and immersive participation. A briefing of how these challenges are addressed is provided in detail in Table 6 (Literature Survey).

## Challenges in traditional telemedicine

Telemedicine improvises accessibility and efficiency but still few challenges need to be addressed i.e. data security vulnerabilities, less patient engagement, scalability, implementation of regulatory compliances, administrative issues etc. The integration of metaverse and blockchain improvises telemedicine by addressing various challenges.

The main challenge in traditional telemedicine practice is securing data and preserving the privacy. Current systems that store EHR centrally face two main security problems: cyberattacks against the servers and data breaches. This research uses blockchain decentralization to develop an encrypted medical record storage system which provides tamper-resistant data protection with precise access permissions. Experiment results confirm that the proposed system blocks unauthorized users from accessing data storage while maintaining compliance with GDPR standards.

Patient engagement is another major problem in the healthcare system. Telemedicine traditionally uses two-dimensional video communication which limits effectiveness of actual physician-patient interactions. In turn it decreases treatment plan adherence from patients. The Metaverse provides three dimensional virtual environments with realistic interactions that enable both consultations, therapy sessions, and rehabilitation interactions. The proposed research for user testing demonstrates that immersive patient consultations duration by 33% while obtaining 4.7/5 points on patient satisfaction reviews.

Administrative tasks may cost extra money and more delays while conducting manual identity verification, managing insurance claims, and patient consent processes. The research uses blockchain smart contracts to produce automates these tasks improvising the efficiency. The implementation of proposed approach has reduced processing time.

The growth of telemedicine solutions also faces a major challenge in scalability since patient data volumes continue to increase steadily. Current platforms experience difficulties in managing their performance levels while new users join. The proposed framework has tried to address this issue by integrating off-chain and on-chain partitioning of the data.

The implementation of digital healthcare systems faces major obstacles when trying to comply with international health regulations such as GDPR, HIPAA etc. Telemedicine systems struggle to resolve legal ambiguities that emerge when patients’ data moves between borders and when healthcare rights need to be handled. The integration of blockchain audit trails with automated compliance checks determines that all data transactions meet the requirements established by GDPR.

In addition, directives, acts, and laws concerned with patients’ rights, freedom of movement, and patient digital identity are not compatible with these technologies. Indeed, to resolve all these issues, it is going to be the question of cooperation between not only the developers of technology tools, but also healthcare organizations, and governmental bodies.

## Proposed model overview

This research proposed an integrated model of telemedicine and Metaverse with Blockchain technology that addresses above mentioned challenges.

- **Data Privacy & Security**: The system promotes data privacy through blockchain-based decentralized storage in addition to managing patient consent through smart contracts which lowers security risks.
- **Patient Engagement**: Patient satisfaction becomes better through Metaverse-based virtual consultations which provide real-time responsive healthcare environment leading to improved adherence from patients.
- **Scalability & Cost-Efficiency**: The Proof-of-Stake (PoS) of Ethereum produces a cost efficient and low latency telemedicine solution.

This research proposes a framework by assessing telemedicine performance which includes:

- Standard benchmarks for evaluating blockchain security in healthcare applications.
- Quantitative scalability and transaction cost analysis.
- Patient engagement metrics in immersive Metaverse environments.

The results of this research enhance next-generation telemedicine systems through systematic solutions which deliver secured, scalable and interactive remote healthcare solutions.

## Literature survey

Telemedicine has undergone significant change over the past several years, mainly due to global crisis such as the COVID-19 pandemic. Current centralized architecture has limitations related to security, scalability, and patient engagement. The literature survey reviews development of traditional telemedicine, blockchain usage to improve security & automation, and the current work on immersive metaverse environments into health sector. The following subsections analyze economic & technical trade-offs, summarize past uses of blockchain in telehealth, and justify reasons why integration of blockchain and the metaverse is necessary.

### Evolution and cost barriers of traditional telemedicine

Telemedicine has finally been embraced as the new way to deliver care, including in areas where supplies are scarce. Its growth was especially boosted by the COVID-19 pandemic that demonstrated the opportunity for delivering consultations in real time, as well as for remotely monitoring and managing chronic conditions^20^. Companies such as Teladoc Health^21^ and Amwell^22^ have shown how telemedicine can be so inclusive in meeting people’s healthcare needs across the world. However, while the telemedicine has emerged as a rapidly advancing field since the COVID-19 outbreak, it remains based on centralized systems, which provokes doubts in terms of data protection, solution’s scalability, and patient involvement.

The implementation of blockchain based telemedicine technology enhances the security, facilitates the automatic processes, and uses decentralized systems^23^. But these factors come with higher costs to deploy. Initial deployment of traditional telemedicine platforms using centralized databases and conventional authentication systems usually costs less but cost will be extended later because of security threats, operational difficulties and regulatory compliance requirements. The comparative cost analysis is briefed in Table 1.

**Table 1:** Comparative cost analysis of traditional vs. Blockchain-Based Telemedicine.

Cost Component	Traditional Telemedicine	Blockchain-Based Telemedicine
Infrastructure Costs^24^	Requires centralized cloud servers, increasing long-term operational costs.	Uses decentralized storage & smart contracts, reducing server dependency.
Security & Compliance^24–26^	High costs due to external cybersecurity audits and HIPAA/GDPR compliance requirements.	Blockchain ensures tamper-proof records, reducing security breach risks.
Transaction Costs	Payment gateways & bank transactions involve third-party processing fees.	Lower transaction costs via blockchain (Ethereum PoS)
Data Storage Costs	Expensive cloud-based EHR storage (AWS, Azure).	(InterPlanetary File System) IPFS/Filecoin decentralized storage significantly reduces costs.
Operational Costs	Requires manual verification of patient data, increasing costs.	Smart contracts automate insurance claims, patient consent, and billing.
Scalability Costs^27^	Expanding requires additional servers and infrastructure upgrades.	Blockchain handles scalability with decentralized nodes without infrastructure overhauls.

**Table 2:** Financial analysis: traditional vs. Blockchain-Integrated telemedicine Costs.

Cost Category	Traditional Telemedicine (USD/Year)	Blockchain-Based Telemedicine (USD/Year)
Cloud Infrastructure (AWS, Azure, Google Cloud)^28^	$100,000 – $150,000 (Traditional telemedicine systems often rely on centralized cloud services, leading to significant annual expenses.)	$50,000 – $80,000 (Decentralized IPFS / Layer 2 Scaling)
Data Security & Compliance^24^	$20,000 (Regular HIPAA/GDPR audits & cybersecurity enhancements)	$5,000 (Blockchain ensures built-in tamper-proof security)
Payment Processing Fees^29^	$30,000 – $50,000 (Bank, PayPal, Stripe fees)	$5,000 – $10,000 (Crypto payments, smart contract settlements)
Operational & Administrative Costs^30,31^	$50,000 – $70,000 (Manual verification & claim processing)	$10,000 – $20,000 (Automated smart contracts for billing & verification)
Scalability Upgrades^32,33^	$50,000 (Adding more centralized servers)	$10,000 (Auto-scaling via blockchain nodes)
Total Estimated Costs	**$250**,**000 – $340**,**000**	**$80**,**000 – $120**,**000**

The approximate analysis as shown in Table 2 includes projected expenses for a medium-scale telemedicine network that handles 10,000 patient visits monthly with two system configurations: traditional telemedicine and blockchain-based telemedicine system.

### Advantages of Blockchain-Integrated telemedicine

Telemedicine systems use blockchain technology with significant investment through smart contract creation and blockchain implementation but eventually generate financial benefits by cutting labour costs, security breaches, and transaction fees. The Table 3 represents the evaluation of how blockchain integration results in substantial financial benefits for extended periods. The cost comparison spread over five years between traditional telemedicine and blockchain telemedicine systems is briefed in Table 3^32–34^. Blockchain administration expenses remain elevated in the first year because of smart contract programming and network configuration however these costs drop in subsequent years because autonomous operating systems. Blockchain lowers operational expenses together with compliance costs because it enables autonomic smart contracts that simplify transaction management and consent procedures while avoiding transaction fees from intermediaries. Lower data storage solution and scalability expenses result from IPFS or Layer 2 scaling methods’ decentralized features. Recognition of cost efficiency and sustainability occurs through blockchain-based telemedicine services which generate $840,000 savings by Year 5.

**Table 3:** Return on investment (ROI) projection for Blockchain-Based vs. Traditional telemedicine Systems.

Year	Traditional Telemedicine (Cumulative Cost)	Blockchain-Based Telemedicine (Cumulative Cost)	Annual Savings with Blockchain
Year 1	$250,000	$150,000 (smart contract development & initial setup)	$100,000
Year 2	$500,000	$200,000	$300,000
Year 3	$750,000	$270,000	$480,000
Year 4	$1,000,000	$340,000	$660,000
Year 5	$1,250,000	$410,000	$840,000

### Comparative analysis of blockchain approaches in telemedicine

With the advent of blockchain technology and its advantages, few approaches are proposed for the telemedicine. These approaches are summarized in Table 4. The table presents an analysis of blockchain-integrated telemedicine services which details key features, strengths, and limitations. Existing blockchain-based systems mainly address data security, interoperability issues, and automated data management systems^35^. The widespread deployment of blockchain-based solutions faces major challenges such as scalability, high computational cost. Most of the approaches lack in real-time processing and standard EHR exchange methods. The table demonstrates that current systems face problems which demand the development of an advanced Metaverse-integrated blockchain framework to handle these issues properly.

**Table 4:** Blockchain-Based telemedicine Approaches.

Works	Key Features	Strengths	Limitations
^35^	Systematic review of blockchain applications in telemedicine.	Identifies potential benefits like data security and interoperability.	Highlight challenges such as data interoperability and regulatory issues.
^21^	Exploration of blockchain’s potential in enhancing telehealth services.	Emphasizes decentralization, tamper-proof data, and improved security.	Discusses adaptability challenges and widespread deployment.
^36^	Proposes SPChain for secure medical data sharing using blockchain.	Enhances data privacy and security in eHealth systems.	Addresses security risks like the 51% attack and challenges in integrating blockchain with existing systems.
^37^	Discusses the integration of blockchain with cloud computing for telemedicine.	Aims to enhance data security and accessibility in telemedicine services.	Potential challenges in system integration and data management.
^38^	Describes a blockchain-enabled telemedicine service developed by Abto Software.	Offers data security, secured access, transparency, and data control.	Requires significant storage capacity and expertise in blockchain technology.
^39^	Discusses the integration of blockchain in telemedicine platforms.	Highlights benefits like data security and improved interoperability.	Implementation complexity and potential changes to existing IT infrastructures.
^40^	Analyzes approaches to integrating blockchain into healthcare data management.	Evaluates advantages and limitations of various blockchain approaches in e-health.	Identifies challenges like stakeholder representation and regulatory compliance.
^41^	Systematic review of challenges in applying blockchain to digital health.	Identifies issues such as regulatory compliance, energy consumption, and data standards.	Emphasizes the need for collaboration among stakeholders to overcome challenges.
^42^	Explores benefits and threats of blockchain as a disruptive innovation in healthcare.	Highlights potential for improved data security and interoperability.	Discusses challenges like energy consumption and regulatory hurdles.
^43^	Discusses various use cases, benefits, and challenges of blockchain in healthcare.	Highlights data security, efficiency, and transparency as key benefits.	Addresses challenges such as implementation complexity and data accessibility concerns.
^44^	Explores how blockchain can enhance healthcare operations.	Emphasizes tamper-proof data storage and improved data interoperability.	Discusses potential challenges in widespread adoption and integration with existing systems.

When Metaverse integrates with blockchain technology for telemedicine it creates a new operational approach that boosts multiple system performance aspects relative to standard telemedicine frameworks and existing blockchain telemedicine systems. A comparative analysis of blockchain-based telemedicine solutions is presented in the Table 5 regarding their performance in security, scalability, cost, interoperability, latency and real-time data handling aspects. The majority of research publications i.e^45–48^. highlight data privacy protection along with decentralized storage as their primary security focus. Practical Byzantine Fault Tolerance (PBFT) consensus addresses scalability doubts by enhancing operational speed^49^ although the problem of system interoperability continues to be futile^50^. The initial blockchain deployment requires technical analysis in terms of costs according to research findings presented in^45^. However, the future operational efficiency of blockchain systems remains underexplored. Only PBFT-based research^49^ examines latency issues and real-time data processing while privacy-based work^51^ analyzes high-latency effects.

**Table 5:** Evaluation of security, scalability, and interoperability in Blockchain-Integrated telemedicine Systems.

Works	Security	Scalability	Cost	Interoperability	Latency	Real-time Data Handling
^45^	Improved data security through decentralized storage.	Addresses interoperability challenges, enhancing scalability.	Implementation may involve significant initial costs.	Enhances data exchange across platforms.	NA	NA
^46^	Enhances security in emergency medical settings.	Challenges in scalability due to interoperability issues.	NA	Challenges in interoperability are briefed.	NA	NA
^47^	Improves security in telehealth platforms.	NA	NA	NA	NA	NA
^48^	Improve security to protect the EHR	NA	NA	NA	NA	NA
^49^	Improves data security and transparency.	PBFT consensus algorithm improves scalability.	NA	NA	PBFT consensus algorithm reduces latency.	PBFT consensus algorithm suitable for real-time data sharing.
^50^	Addresses data privacy on the Internet of Medical Things (IoMT) for remote healthcare.	NA	NA	Addresses interoperability barriers in IoMT.	NA	NA
^51^	Enhances data privacy using blockchain.	NA	NA	NA	Observed high latency	NA
^52^	Addresses security and privacy challenges in IoMT.	NA	NA	NA	NA	NA
^53^	Enhance security by using machine learning and blockchain	Addressed scalability using machine learning	Addressed cost using machine learning	Addressed interoperability issues	NA	NA
^54^	Enhance security by using Augmented Intelligence of things (AIoT) and blockchain	Addressed scalability	Computational cost reduction is achieved	NA	Latency is addressed by automating processes	NA

* NA: Not Addressed.

### Integrated Metaverse-Blockchain for telemedicine

The discussed challenges can be addressed with metaverse and blockchain as mentioned in Table 6. Telemedicine is a method for delivering healthcare by using electronic communication and information technologies. Metaverse provides a more engaging environment for patients^55^. Key benefits include:

- **Enhanced User Engagement**: Creating a realistic sense of presence between patients and health care providers seems to be easier with 3D interaction. This is because there are emerging references that use of such environments improves patient satisfaction and compliance with medical domains^56^.
- **Virtual Training and Education**: The Metaverse is perfect for simulation-based learning, which is important to healthcare stakeholders in practice. This minimizes the use of the physically presence and eliminates barriers of geography^57^.
- **Remote Monitoring and Rehabilitation**: Patients can receive primary care, physiotherapy sessions, through reliable online ways, which promotes convenience and decreasing the need for journeys^58^.

Blockchain addresses critical concerns in telemedicine through its decentralized and secure nature:

- **Data Security and Privacy**: Blockchain makes data secure and puts patient record data on the block where no one can tamper with it without authorization. This ensures no risks which can be affected by the centralized management of data.
- **Smart Contracts**: Other classical activities like consent management, EHR access, and insurance claims also have less paperwork and are made more efficient.
- **Interoperability**: One of Blockchain’s main advantages is that the data can always be shared between the platforms following a standardized protocols to facilitate the interoperation and coordination of care.
- **Auditability**: The nature of blockchain makes transactions evidence based, and this characteristic is well best suited to the health sector due to the unique nature of transactions involving its data.

**Table 6:** Challenge mitigation using metaverse and Blockchain.

Challenge	Description	Impact	Solution via Metaverse	Solution via Blockchain
Data Security	Centralized storage impacting breaches and unauthorized access.	It results in patient negligence, the loss of money, and fines.	Ensures confidentiality	Minimizes the possibilities of unauthorized access & decentralized storage
Patient Engagement	Lack of physical touch distancing lowers the overall satisfaction level.	Lower treatment effectiveness especial in mental illness and chronic conditions.	Virtual, 3D consultations	Ensures monitoring of conformity and approval as well as in a way in which it will be easy to validate and demonstrate.
Interoperability	Data interoperability	Delay in care co-ordination, and patients missing part of their medical records.	Real time data sharing and visualizations	integration between platforms that use harmonized blockchain agreements for data sharing.
Scalability	Increased demand causing infrastructure bottlenecks and delays.	Reduced quality of service and patient dissatisfaction.	Scalable virtual environments	Manages large-scale transaction logs efficiently with decentralized ledger systems.
Privacy Concerns	Patients worry about how their sensitive health data are collected, stored, and used.	Reluctance to adopt telemedicine and mistrust in healthcare providers.	Ensures private, avatar-based interactions that mask user identities when necessary.	Provides immutable audit trails and fine-grained access control using smart contracts.
Technological Access	Limited access to high-speed internet and devices in rural regions.	Excludes underserved populations from benefiting from telemedicine.	Lightweight virtual spaces optimized for low bandwidth	Employs off-chain storage solutions for bulk data, reducing reliance on high-speed internet.
Legal and Compliance	cross-border telemedicine practices.	Hinders global telemedicine adoption	Geo-specific virtual environments tailored to regional regulations.	Maintains tamper-proof records that comply with global standards like GDPR and HIPAA.
Cost Efficiency	High operational costs for telemedicine platforms, including infrastructure and staffing.	Limits scalability and affordability for providers and patients.	Reduces physical infrastructure training facilities.	Automate processes such as claims and billing, reducing administrative costs.
Trust and Accountability	Lack of transparency in billing, treatment plans, and consent processes.	Mistrust between patients and providers leads to lower adoption rates.	Real-time, interactions in virtual environments.	Transparent tracking of transactions and actions in healthcare using blockchain ledgers.

## System architecture and layered interaction

**Fig. 1:**
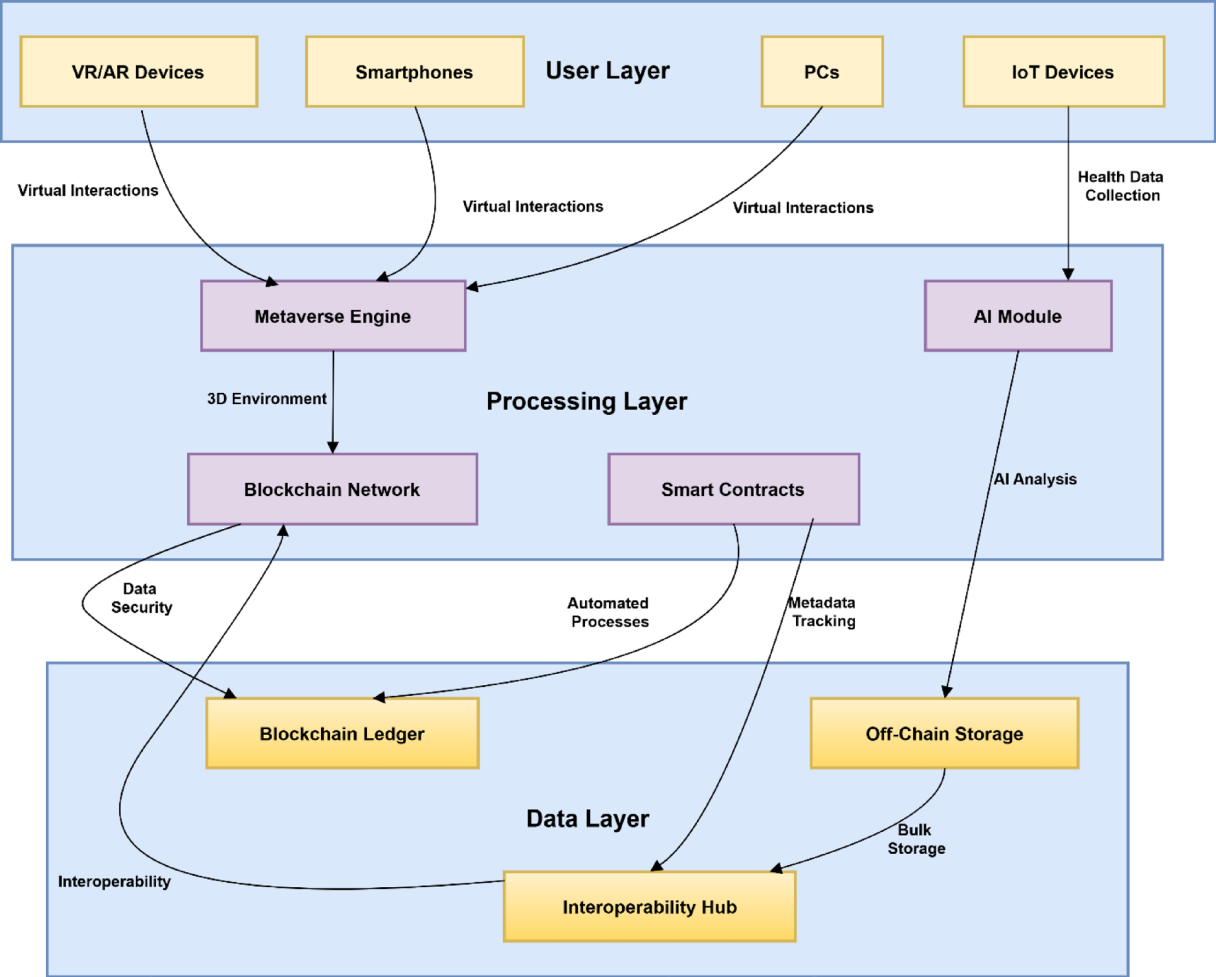
Architecture for telemedicine with integration of blockchain and metaverse.

After analysing the research gaps in telemedicine domain, this research proposed novel approach for the telemedicine functionality by using blockchain and metaverse as mentioned in Fig. 1.

The components of the architecture are as below.

### User layer

This layer is one which enables users- patients, healthcare providers, and others to get an interface to the system. It includes the following components:

#### Virtual reality (VR)/Augmented reality (AR) devices

VR / AR devices i.e. Oculus Rift, HTC Vive or the Microsoft HoloLens can be used. These are used to build a three-dimensional realistic environment for consultation, therapy, and especially in medical training. These devices assist in increasing patient interaction by providing an environment in which patient interactions can be scheduled.

#### Smartphones

For patients who do not have advanced equipment, smartphones act as the first gateway into telemedicine. Patients can endorse consultation and monitoring through mobile handsets. This makes them a kind of middle ground between such systems based on ordinary telemedicine and those connected with the Metaverse.

#### PCs

Laptops, notebooks and tablets used in both clinical and administrative settings to access the patients’ information, engage in virtual environment, and organizing work. It also applies when doing complex models such as 3D anatomical models or real time diagnostics.

#### Internet of things (IoT) devices

IoT devices include smart health devices for usage in health monitoring, connected medical devices, and smart home health systems. These devices can obtain accurate real-time health parameters (heart rate, blood pressure, glucose level etc.) and pass on the data to the processing layer. All of them enable ‘outpatient’ patient care, as well as inform decisions based on objective, administrative data inputs.

### Processing layer

This layer deals with the main functions like generating environments, securing information, and the performance of the necessary healthcare workflows.

#### Metaverse engine

The engine drives a three-dimensional virtual environment for the patients’ communication with the provider. It permits virtual clinics in which consultations, therapies or even surgery can be carried out. It enables the health practitioners to practice real-life experiences in the lessons they have learnt.

#### Blockchain network

It is distributed ledger technology that ensures all data transmittals in the telemedicine context are safe. It supports confidentiality, accountability and traceability of patient’s information. It also enables transfer of information across these distinct platforms while avoiding vulnerability to third party interference. Every access and transmission of the health information is recorded on the blockchain through the consensus algorithms. Use case opt for any blockchain platforms as per the requirement of the system. Depending on the blockchain platform, consensus algorithm may vary as shown in Table 7. System has opted PoS considering the energy efficiency, high scalability, and public healthcare systems. As Ethereum is used in the proposed approach, PoS is used in the experimentation.

#### Smart contracts

Smart contracts i.e. programmable logic resides on the blockchain platform which gets executed automatically after the trigger. It involves actions like, patient consent confirmation, insurance reimbursement, EHR access, and charges. Smart contracts cut down on expenses of numerous administrative procedures and enhance system functionality.

**Table 7:** Comparison of consensus algorithms with respect to the healthcare.

Consensus Mechanism	Description	Advantages	Limitations	Suitability for Healthcare
Proof of Work (PoW)^59^	Nodes solve complex cryptographic puzzles to validate transactions (e.g., Bitcoin).	High security, decentralized.	Slow transactions, energy-intensive, costly.	Not suitable due to inefficiency.
Proof of Stake (PoS)^60^	Validators are chosen based on the amount of cryptocurrency staked.	Energy-efficient, faster than PoW, scalable.	It can be prone to centralization if few stakeholders dominate.	Suitable for public healthcare blockchains.
Proof of Authority (PoA)^61^	Transactions are validated by pre-approved nodes (trusted authorities).	High speed, low computational cost, efficient for permissioned networks.	Requires trust in selected validators, less decentralized.	Best for private healthcare blockchains.
Delegated Proof of Stake (DPoS)^62^	Stakeholders vote for delegates who validate transactions.	Fast, scalable, energy efficient.	Some centralization risks due to small validator set.	Good for healthcare consortiums.
Byzantine Fault Tolerance (BFT)^63^	Consensus is reached through agreement between known nodes.	High security, fast, reliable.	Limited scalability, complex implementation.	Used in Hyperledger Fabric for healthcare.
Proof of Elapsed Time (PoET)^64^	Uses a trusted execution environment (TEE) to assign wait times to nodes before granting consensus.	Energy-efficient, fair selection process, scalable.	Requires hardware support (Intel SGX), not fully decentralized.	Suitable for permissioned healthcare networks needing efficiency and security.

The smart contracts are modular and version-managed which allows for simple patches via updates. There are critical fallback () functions in contracts and a privileged account can execute them. If problems are found, then sensitive tasks are momentarily suspended. Every Ethereum event helps keep a track of all transactions and operation. When a smart contract is failed then, these logs allow steps in the process to reset operations during recovery or migration. Since EHR records are stored in IPFS, not on Ethereum’s chain, they retain their integrity even when the smart contract is failed.

**Pseudocode of the smart contract used in the system is represented below.**


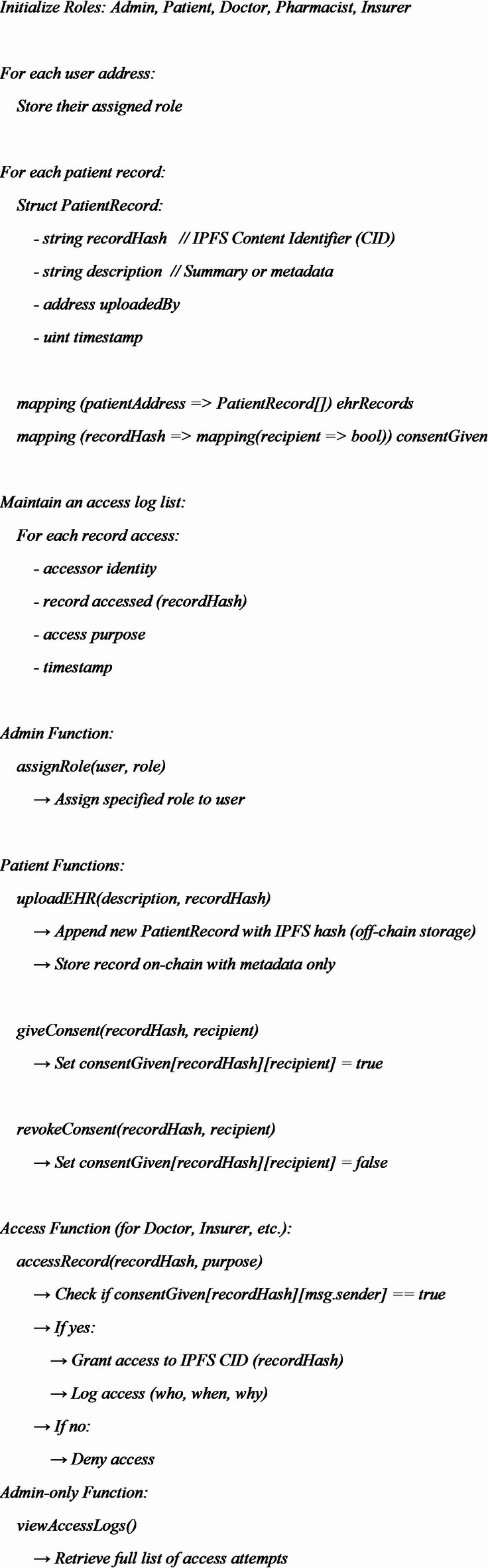


With Ethereum smart contracts, the architecture uses Role-Based Access Control (RBAC) to ensure legitimate data permissions. An admin assigns the roles as Patient, Doctor, Pharmacist, Insurer and Admin to different users using the assignRole() function. As per the roles, access privileges can be set up to access EHR.

EHRs is owned by patient. A patient’s updated record is first encrypted, then the IPFS hash of the record along with a metadata like timestamp, addresses etc. are uploaded on-chain using the uploadEHR() function. Thus, sensitive information stays safe and protects privacy.

Patients have to give their consent before an EHR can be accessed. Using the giveConsent() and revokeConsent() functions, patients can control access privileges to EHR. An on-chain two-dimensional mapping ensures that IPFS record hashes are linked to the receiver’s addresses. This makes certain that access is specific and can be managed and changed by the patient.

The system uses the accessRecord() function to ensure that consent has been granted before another stakeholder such as a doctor or insurer, tries to access a patient’s record. If authorized, the system gives access to the concerned IPFS hash and at the same time logs it with the accessor’s identity, reason for accessing and the time it happened. Whenever there is no consent or the consent has been withdrawn, default access denial makes sure no unauthorized login occurs.

The system logs all access events on the blockchain and the admin can view them by using the viewAccessLogs() function. The trail of events in the audit proves trustworthiness, helps with reporting and demands responsible handling of medical data.

#### AI module

This module is designed to apply machine learning for operational data analysis on patient’s data and to produce tangible results. It also enforces prediction of various conditions, diagnosis, treatment, and care plans at an individual level. It analyses IoT data to identify either a device malfunction or a threat to the patient’s health in real time. Isolation Forests is used in the module for detecting the anomalies in IoT and health telemetry data. The system focuses on data balancing and continuous monitoring to reduce bias.

### Data layer

This layer is one that maintains complete control as well as the handling of storage, transfer, and management of these data.

#### Off-Chain storage

The storage saves large databases including, for instance, medical images, EHRs, and therapy videos which are not realistically feasible to store on the blockchain. Scalable and cost-effective solution is IPFS same as in some cloud solutions. Referring to the use of metadata, it is safe to say that metadata is connected to blockchain.

Proposed system relies on IPFS to securely and confidently store EHRs using content-addressed hashing. Standard IPFS does not have encryption mechanisms. To solve this problem, the proposed system first encrypts every medical record with AES-256 and then adds them to IPFS. Only resulting CID is stored on-chain. IPFS stores medical records securely against the unauthorized access, data loss, and tempering with the help of integration of encryption, governance via blockchain, and distributed chunks of EHR on IPFS.

#### Blockchain Ledger

The ledger records and holds data that pertains to patient, conversation and sales records. It also makes all actions undertaken in the system accountable and immune to alteration by unauthorized persons^65^. It has an added advantage of offering a compliance to regulations such as GDPR and HIPAA.

#### Interoperability hub

It Enables smooth interoperability via data sharing between various telemedicine systems, care givers and other applications. This way it provides compliance with worldwide healthcare data standards such as Fast Healthcare Interoperability Resources (FHIR). It also fosters integrated care by providing coherent approaches to the accessing of data pertinent to patients.

Confidentiality of sensitive health information is maintained in the proposed architecture as mentioned below.

- **Off-chain Encrypted Storage**: EHRs, reports and therapy information are first encrypted by AES-256 before being put into IPFS. A cryptographic hash called the CID is all that gets recorded to the Ethereum blockchain to guarantee patient data are kept hidden.
- **Smart Contract-Based Access Control**: Access is granted to medical data using smart contracts that after patients have agreed. All access requests are kept on the blockchain so they can be checked and verified.
- **Minimal On-Chain Exposure**: No personal info such as name or address can be found on the blockchain. Just the timestamp, access hash and type of record are logged by the system.

### Interaction across the layers

The system has three main parts: the User Layer, Processing Layer and Data Layer, each one intended to deliver secure and immersive telehealth services.

VR/AR devices, IoT wearables and both mobile and desktop applications are part of the User Layer where patients and doctors communicate. Here, real-time data are generated with the help of different movements, voice, and sensor devices.

All this information is passed to the Processing Layer, where it is performing following tasks.

- Delivering the realistic 3D virtual meeting space using Metaverse Engine.
- Making sure transactions are validated and data logs are uploaded to Blockchains.
- Initiating Smart Contracts to ensure consent and billing.
- Analysing sensor data into predictions of current conditions and early diagnosis using Artificial Intelligence (AI) algorithms.

Once processing is done, data are sent to the Data Layer, where EHRs, diagnostic images and therapy records are stored off-chain. Assets in a blockchain are given timestamps, access restrictions and digital hashes that are stored permanently.

Communication across these layers is facilitated through security-focused Application Programming Interface (APIs). As an example, data on a patient’s vitals from the User Layer are examined by the AI Module (Processing Layer) and the main discoveries are saved in the Data Layer. Likewise, if a doctor issues a treatment plan through the Metaverse interface, a smart contract (Processing Layer) updates the patient’s data in the blockchain (Data Layer) and sends an instant message back to the patient. As a result of this structure, messages respond in real time, records are secure and shared across different platforms, following the FHIR data standards.

Unity 3D is used in the system for building a fully immersive experience. Ethereum is used to make sure transactions are secure and users are managed with smart contracts. Unity 3D, is deployed using Web Extended Reality (WebXR) and Unity Web Graphics Library (WebGL), facilitate 3D interaction between patients and doctors. Scripts for Unity were used to show how a doctor and patient would interact, how records are accessed and what treatment areas are like. On the server, Ethereum Sepolia is used as a testnet via Web3.js for identity verification, fetching health records and audit logging. Role-based access control, patient consent management and record access were handled by smart contracts in Solidity. The metadata was stored on the blockchain, while the full EHR files were uploaded to IPFS. By merging Unity for the front-end with Ethereum for backend functions, the system’s telemedicine experience guarantees security, decentralization and engaged telemedicine experience.

## Experimentation and results

The proposed telemedicine system based on Blockchain and Metaverse technology integrates VR / AR, IoT, and AI for enhancing telemedicine solutions. The system architecture includes four essential layers which includes User Interaction, Data Processing, Blockchain, and Storage & Interoperability layers. The system incorporates these layers that enable better patient engagement, protects medical data, facilitates seamless interoperable EHR sharing, and automates healthcare functions.

### System configuration

#### Metaverse & VR/AR for telemedicine consultations

A VR/AR system enables the User Interaction layer to develop realistic virtual meeting spaces where doctors interact live with patients for clinical consultations. The development of 3D virtual environments is done with Unity 3D and Unreal Engine. WebXR API provides compatibility with browser-based applications that work for users who don’t have dedicated VR devices. The system uses Photon Engine together with real-time functions for multi-user support that enables virtual meetings between several patients and doctors during consultations and therapy sessions.

Patient engagement becomes enhanced through avatar-based interactions which allow patients to produce digitized versions of their selves. Virtual consultations gain better realism through patient and doctor gesture tracking which utilizes Leap Motion devices and Oculus Hand Tracking SDK sensors. Security and privacy are achieved through Advanced Encryption Standard (AES)-256 encryption of virtual interactions and consultation data which then gets stored in IPFS.

VR-based telemedicine technology functions best when there are low-latency interactions below 20 milliseconds and requires bandwidth between 10 and 50 Mbps which depends on the level of complexity present in the virtual environment. The NVIDIA RTX series of GPU-accelerated hardware serves the best for efficient rendering of 3D models alongside avatars.

#### IoT-Driven health monitoring and AI-Based diagnostics

Data Processing Layer integrates medical IoT devices and AI-driven health analytics to provide real-time patient management and diagnostic details. Medical-grade electrocardiogram (ECG) / Blood Pressure (BP) monitored devices, Apple Watch, and Fitbit devices record vital measurements consisting of heart rate, blood pressure, oxygen level, glucose levels etc. in real-time. The system uses (Message Queuing Telemetry Transport) MQTT protocol and Bluetooth Low Energy (BLE) to establish live data transmission with the system.

Convolutional Neural Networks (CNNs) and Long Short-Term Memory (LSTMs) detects anomaly detection on patient health data. Healthcare data enabled this system to receive real-time deployment through TensorFlow Serving while it identifies arrhythmias or sudden oxygen level drops. Real-time medical data are processed by the system to achieves data processing speeds lower than one second which enables doctors to receive immediate emergency alerts.

The system enhances scalability through Kubernetes-based auto-scaling that maintains support for more than 10,000 connected IoT devices in the data stream. The system integrates Edge computing devices including Raspberry Pi to perform AI inferences on the device which lowers the need for constant cloud connectivity.

#### Blockchain for security, smart contracts, and data integrity

Data integrity, data security, and automation of healthcare processes is handled by Blockchain Layer. The system is implemented on Ethereum 2.0 that uses a PoS consensus protocol for maximizing scalability and minimizing power consumption. The Solidity-based smart contracts accomplish key healthcare operations that handle patient consent management, EHR access, or insurance claims management.

The system maintains the data privacy through SHA-256 hashing which stores EHR cryptographic hashes on-chain but stores actual EHRs together with large files within the IPFS as off-chain. EHR access is controlled by the patient through the smart contracts. The blockchain infrastructure enables HIPAA and GDPR compliance thus providing security measures for patient data protection as well as regulatory compliance.

With perspective of the feasibility, Ethereum 2.0 utilizes PoS to reduce transaction costs as compared to PoW along with providing better than 10,000 Transactions Per Second (TPS) throughput. The blockchain meets requirements for performing large-scale telemedicine operations due to its design features.

This choice was based on requiring a blockchain infrastructure that could grow, be energy efficient and be used worldwide. Because it is a public blockchain, permissionless access is possible in which is not possible in private blockchains like Hyperledger Fabric. Permissionless access facilitates participation across countries in telemedicine networks. As consensus used is PoS, the cost and energy needed for transactions have fallen, while keeping the network secure and decentralized.

Also, there are many tools and resources for developers on Ethereum which made it easy to connect smart contracts, wallets (like MetaMask) and IPFS. With these integrations, data immutability, smart contract based automated consent, and legitimate data access is possible without a centralized trust anchor.

Hybrid and private blockchains perform better, but they do not assure enough transparency or the scalability which are necessary for continued success in health data platforms.

#### Feasibility and scalability analysis

The system has addressed common challenges i.e. scalability, security, and performance, which are generally observed in telemedicine applications. The following factors were considered for this proposed system’s implementation.

- **Throughput**: The system operates with a capacity to manage 10,000 active IoT data streams while executing blockchain transactions at above 10,000 TPS.
- **Cost Efficiency**: The Ethereum’s PoS consensus mechanism and EHR partitioning as on-chain and off-chain decreased the cost significantly.
- **Regulatory Compliance**: The implementation of blockchain audit trails with access control adheres to the regulations GDPR standards.

#### Latency optimization strategies

Low latency from the system is necessary so that telemedicine works smoothly when using VR technology. Proposed framework achieves low latency with front-end optimized rendering and decentralized data control.

With WebXR, the User Layer makes it possible for clients to have immersive telemedicine consultations quickly with minimal overhead. WebXR’s asynchronous rendering and ability to interact across a variety of devices, latency of interactions is always below the 50 ms.

Real-time data from sensors and media streaming are enabled using MQTT and Web Real-Time Communication (WebRTC) at the communication layer of the system. Because the protocols are both triggered by events and require only a little bandwidth, they work well with low-resource environments.

Besides, health information like EHRs and diagnostic details is not directly stored on the blockchain, but only their metadata and hash values are stored. EHRs and diagnostic details are stored off-chain i.e. on IPFS. This helps reduction in congestion when sending transactions and lets the system complete them in low latency.

By creating a system from these features, the framework handles responsive and flexible telehealth with low latency real-time experience. Integrating these platforms helps provide a seamless workflow for delivering decentralized telehealth services. When patients enter into VR, they are verified using smart contracts on Ethereum. Medical records are safely accessed from IPFS by looking up the hash on the blockchain. When consultations take place, IoT devices deliver health data that the AI module examines and flags any suspicious data, offering support for diagnostics in the virtual platform. Such integration of Metaverse, Blockchain, IPFS, and AI collaborate to create a telemedicine system that is responsive and secure.

### Results

Performance was measured for the system by using controlled simulation and comparing it to benchmarks. Experiments using WebXR showed that latency stayed under 50ms every time, reaching the levels required for VR in industry consultations. Data recovery from IPFS was analysed during 100 concurrent users and the average access latency was below 300ms. Transactions involving smart contracts on the Ethereum Sepolia testnet, with web3.js, took an average of 0.45 s to execute. Scalability was assessed based on Node.js which was used to check the system during parallel sessions of up to 1000 users. Transaction latency, throughput, success rate of sessions and satisfaction scores were determined through logs, performance of smart contracts and responses from users. All testing involved running synthetic workloads for a number of people ranging from 100 to 10,000 and internet speeds that varied from 0.5 to 5 Mbps. All results in the figures below are from groups of samples that were tested an average of three times on controlled equipment.

#### User engagement

It quantifies the extent to which the system captures user interest and maintains compliance with therapy plans. It consists of number of visits per user, length of visits, patient reported satisfaction and success rates of the users in continuing to use the website or app. Telemedicine solutions depend heavily on active user participation because this determines both patients keep rates and satisfaction scores. Following factors were considered for this evaluation:

- **Session Duration**: Average amount of time spent by the patient during their virtual consultations.
- **Patient Retention**: The frequency of user returning for follow-up medical appointments.

**Fig. 2:**
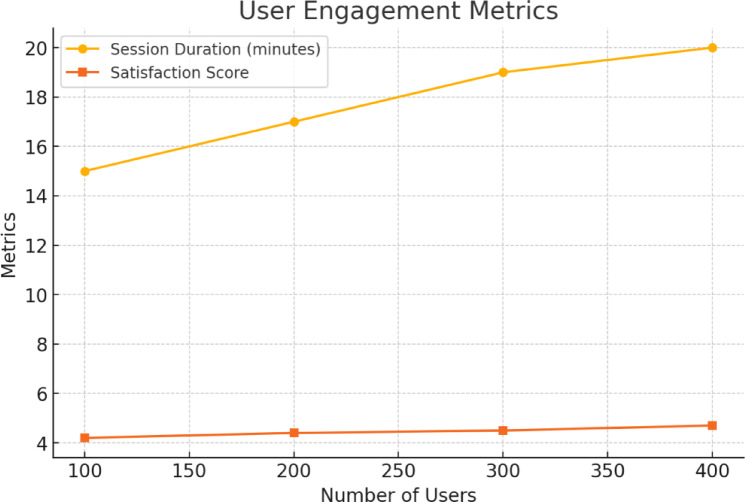
User engagement with the system.

Using Fig. 2 below, the satisfaction score was recorded for the users. The system was tried out on a local testbed using WebXR-enabled 3D consultation rooms. A series of simulation sessions took place for 100, 200, 300 and 400 users with scripted actions and session logging. Data on session duration and patient satisfaction was collected from the feedback forms. This shows how people use telehealth platforms that are based on a metaverse. The frontend was built using Unity WebGL and the backend metrics were logged using Flask and its integration with Firebase.

#### Data security

**Fig. 3:**
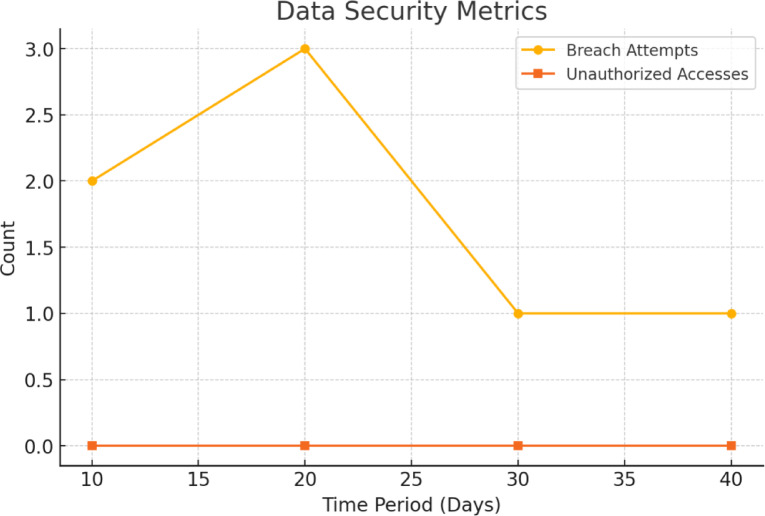
Access Management to the system.

It records the occurrences of data loss or violation or unauthorized access attempts. Blockchain will make the data very secured by encrypting it and at the same time the records cannot be altered. The system was evaluated based on number of detected unauthorized login attempts and the total number of intrusions attempts that blockchain security system successfully obstructs. The research conducted simulated penetration tests for cybersecurity assessment of blockchain EHR permission systems. Smart contract-based RBAC was used in this system. Notably, as it will be observed from Fig. 3, unauthorized access is not permissible in the system. Security testing was performed by trying out unauthorized access methods using HTTP and smart contract API functions. Penetration test was performed using manual test scripts to trigger invalid results based on roles. All access to the blockchain was handled via RBAC with logs of all unauthorized actions. Attempts to breach the network were found by looking at flags in the event logs produced by Ganache.

Although these simulations provided the initial insights, more comprehensive testing using frameworks such as Open Web Application Security Project (OWASP), MythX or Slither will be done in future research.

#### Efficiency

**Fig. 4:**
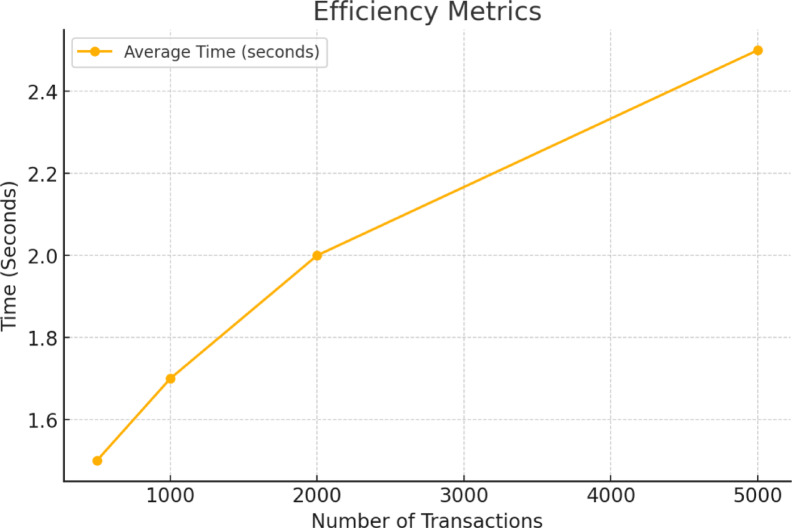
Efficiency of the system.

System measures the efficiency on time and accuracy during tasks such as consultation time, data search, and claims. The system is evaluated with time taken for confirmation of blockchain transactions and rendering 3D environments with syncing of real time transactions. As depicted in Fig. 4 above, with increase in the number of transactions is inversely proportional to the time taken for the actual execution.

To check efficiency, batches of transactions (from 500 to 5000) were submitted to the testnet and recorded their average transaction confirmation time. Every batch performed activities such as consent updation and granting access to the medical record. Web3.js scripts were used to send transactions and record timestamps at both initiated and confirmed stages. Latency was averaged for each batch size based on results from 5 different runs.

#### Scalability

**Fig. 5:**
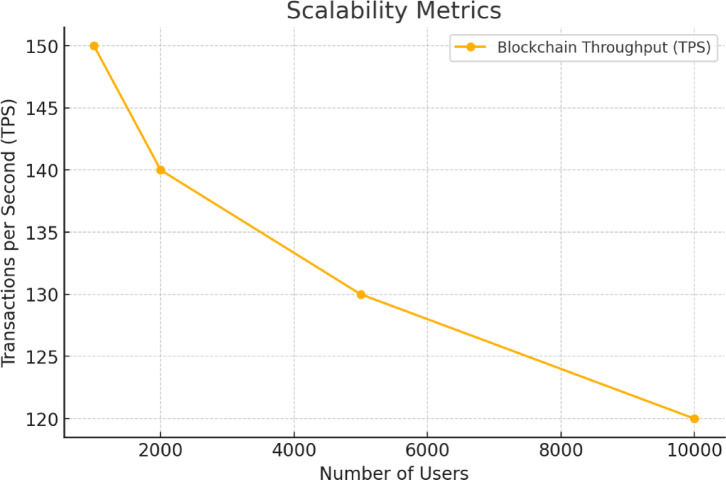
Scalability of the system.

This metric evaluates the capabilities of the system in terms of loads including the number of users at any one time or the number of transactions per block. The system was evaluated with maximum number concurrent users and throughput of the transaction in TPS. The system was tested at 1000, 2000, 5000, and 10,000 simulated concurrent users. As depicted by Fig. 5, when number of users increases the throughput of the system reduces.

Scalability was evaluated by sending different numbers of concurrent transactions with simulation of 1000 to 10,000 concurrent users. Apache JMeter and custom load generation scripts were used to dispatch transactions simultaneously and count all successful commit. Under each different user load scenario, the TPS was measured over a 1-minute interval.

#### Accessibility

With the extent of the platform and how effectively it can be accessed especially in developing nations describes accessibility. Examples of the metric include the rate of sessions that has been accomplished under conditions of low bandwidth.

**Fig. 6:**
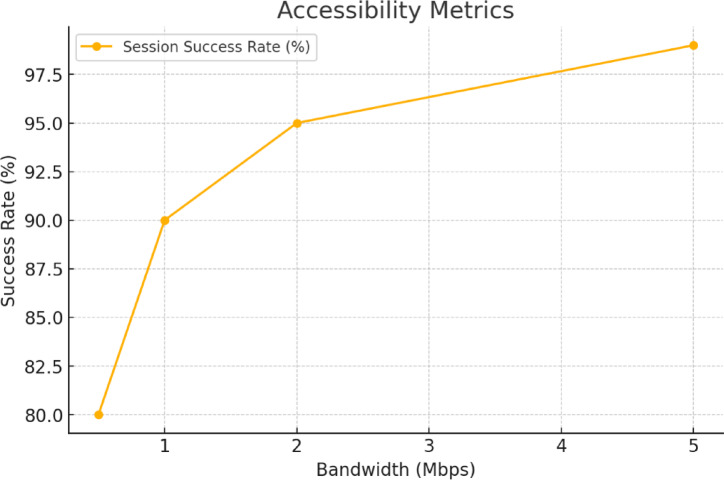
Success rate of the system.

It can be observed from Fig. 6 that as the bandwidth is increased then the success rate also increased. By controlling internet speed with NetEm, WebXR-based consultation environments were simulated operating with bandwidths from 0.5 Mbps to 5 Mbps. When the user was able to perform following activities, it was considered as a successful session.

- Launch the environment for the consultation.
- Fetch EHRs stored on IPFS using the Ethereum blockchain.
- Perform system tasks for at least 5 consecutive minutes without any troubles.

The test scenarios ran 3 times, and the average success rate was recorded. The use of this method shows how real conditions in rural or isolated medical care can influence data transmission.

### Comparative evaluation with existing Blockchain-Based telemedicine approaches

Although blockchain is used in healthcare to improve security and the metaverse for improved immersive training and patient interaction, very few research work is done in integration of both technologies. It is observed from reviewing the open-access and peer-reviewed studies that the use of blockchain and metaverse in telemedicine is not fully explored, specifically with measurable empirical results.

This gap is addressed with comparison of our proposed system against three notable publications^66–68^. These articles offer models or blockchain telehealth systems, but they lack experimental tests and immersive scenarios.

**Table 8:** Comparative analysis with proposed Approach.

Metric	Proposed System	^66^	^67^	^68^
Session Latency	< 50 ms (WebXR)	Not specified	Not specified	Not specified
EHR Access Time	< 300 ms (IPFS)	Not specified	Not specified	Not specified
Transaction Confirmation	0.45 s (Ethereum Sepolia)	Not specified	Not specified	Not specified
User Scalability	Up to 10,000 users (JMeter)	Conceptual only, not quantified	Not specified	Blockchain scalability mentioned, no user count given
Satisfaction Score	4.7/5	Not measured	Not measured	Not measured
RBAC Security Logs	Enabled, 0 unauthorized access	Mentions basic access controls, no RBAC log stats	Access control discussed but no performance results	Security features conceptual only
Real-time Environment	WebXR + Unity WebGL	Web-based telemedicine interface (2D), no XR	None mentioned	None mentioned

In Table 8, the performance of each article is compared regarding session latency, EHR access time, blockchain transaction confirmation, scalability, user satisfaction, security, and support for real-time environments. The proposed system is unique one to offer a fully built system and tested with WebXR, IPFS, and smart contracts on a testnet.

### Real-World implementation challenges

Though proposed system is technically capable and succeeds in simulation, there are challenges to implementing it in real life:

- **Regulatory and Compliance Barriers**: Deployment of system in different countries, it must be ensured that system adhere to compliances like HIPAA, GDPR and laws concerning to that country. One of the guidelines of such compliances i.e. “Right to be forgotten” is difficult to implement as blockchain stored data are immutable.
- **Infrastructure Disparity in Rural Areas**: It is very hard for underserved locations to have high-speed internet, use compatible XR devices or have secure local nodes. This problem limits fair use of telemedicine, especially in areas where lower bandwidth or obsolete hardware is prevalent.
- **Integration with Legacy Systems**: Hospitals are still mostly relying on EHRs systems those are not interoperable. Even with FHIR standards usage, seamless and secure integration is difficult because of varying data formats and access protocols.
- **User Learning Curve and Trust**: Both patients and doctors may take time to learn and use immersive XR interfaces and blockchain-based tools.

## Conclusion

This research provides a solution to the problems associated with traditional telemedicine through the use of the Metaverse and blockchain. This research presents a Metaverse-Blockchain integrated telemedicine model that addresses the security, scalability, interoperability, and engagement challenges in traditional remote healthcare solutions. This research fills the gap in the existing literature and contributes to the stream of digital health and novel technology by underlining the ability to reshape telemedicine with secure, scalable, and immersive solutions. The proposed system provides the frameworks to strive for achieving fair, optimal and patient-centric driven healthcare delivery in the context of the emerging digital world. The research states that blockchain enhances healthcare system by creating immutable patient centric decentralized systems which reduced unauthorized data access attempts. The healthcare automation based on smart contracts enables patient consent management, insurance claims management, and secure transactions while reducing administrative costs and improving operational efficiency.

The experimental evaluation using the set of metrics substantiates the proposed system and provides a standard for future applications. The evaluation of system confirms that the proposed system has improvised the data security, with reduced costs and higher scalability than traditional telemedicine. The blockchain has undergone for performance testing at different user-load levels to demonstrate low latency and higher throughput. The cost analysis demonstrates that distributed blockchain data storage method decreases long-term financial demands which makes large-scale system adoption financially achievable. The proposed research establishes foundations to develop blockchain-based secure telemedicine solutions with user-friendly Metaverse technology while preserving patient privacy. This research solves main security problems thus advancing digital healthcare technologies into the future generation.

## Future work

While this proposed telemedicine system integrates Blockchain and the Metaverse successfully, it still has several limitations:

- **Test Environment Limits**: The evaluation was performed within a controlled test environment that used WebXR, Unity WebGL, Ethereum Sepolia testnet, and IPFS nodes located locally. Real variations in internet speed, device capabilities, and network congestion, may differ from the test results.
- **Scalability Beyond 10**,**000 Users**: Though 10,000 virtual users were tested, how the system could scale in real scenarios under more stress is still unclear. More studies on fully decentralized networks must be done.
- **Limited Security Testing**: In this case, all penetration testing was done manually. Instead, tools like OWASP Zed Attack Proxy (ZAP), Slither or MythX are required to evaluate resistance against vulnerabilities in smart contracts and breaches.
- **Interoperability with Existing EHR Systems**: Although the framework adopts FHIR, no real tests of integration with hospital information systems have been done.

## Data Availability

Data will be made available on request to corresponding author.
